# LL37-Derived Fragments Improve the Antibacterial Potential of Penicillin G and Ampicillin against Methicillin-Resistant *Staphylococcus aureus*

**DOI:** 10.3390/antibiotics12091398

**Published:** 2023-09-01

**Authors:** Wenxu Han, Terri A. Camesano

**Affiliations:** Department of Chemical Engineering, Worcester Polytechnic Institute, 100 Institute Road, Worcester, MA 01609, USA; whan@wpi.edu

**Keywords:** methicillin-resistant *Staphylococcus aureus*, antimicrobial peptides, synergistic combination, penicillin G, ampicillin, re-sensitization

## Abstract

Methicillin-resistant *Staphylococcus aureus* (MRSA) infections are a severe threat to public health. Antimicrobial peptides (AMPs) are novel and potential antimicrobials with specific antibacterial mechanisms. Our aim was to study the potential of LL37, FK16, and FK13 to enhance the anti-MRSA activity of antibiotics in vitro, particularly penicillin G and ampicillin. Our results showed that FK16 and FK13 have more synergistic inhibitory effects to MRSA strains when combined with penicillin G and ampicillin. In addition, AMPs exhibited strong membrane permeabilizing properties, and membrane permeabilizing effects can provide a possible explanation for the improved antibacterial effects of antibiotics, since permeabilizing AMPs have the potential to increase the access of antibiotics. To further study the electrostatic interactions among cationic AMPs with negatively charged bacteria, we measured the zeta potentials of three MRSA strains and also neutralized three MRSA strains with the addition of cationic AMPs. Further, we demonstrated the connection between membrane permeabilization and zeta potential neutralization. Finally, we treated MRSA strains with AMPs and characterized the MICs of penicillin G and ampicillin. FK16 was the most promising AMP among the three AMPs, since exposure to FK16 decreased the MICs of both penicillin G and ampicillin for all MRSA strains and also demonstrated more synergistic combinations when combined with antibiotics. AMP exposure and subsequent membrane permeabilization provide a possible pathway to re-sensitize drug-resistant bacteria to traditional antibiotics. Re-sensitization may help preserve the effectiveness of traditional antibiotics, thus providing a potential new strategy for fighting MRSA infections.

## 1. Introduction

The World Health Organization (WHO) considers methicillin-resistant *Staphylococcus aureus* (*S. aureus*, MRSA) a priority pathogen, which severely threatens public health [1]. Many severe infections such as pneumonia, bloodstream infections, and surgical site infections can be related to MRSA, and these MRSA infections can be fatal for patients [2]. The Centers for Disease Control and Prevention (CDC) estimated that more than 70,000 severe infections and 9000 deaths can be attributed to MRSA each year [3].

MRSA is resistant to almost all β-lactam antibiotics as these strains express a non-native penicillin binding protein (PBP) called PBP2a [4]. PBPs play important roles in cell wall synthesis, and β-lactam antibiotics can achieve antibacterial effects through binding with PBPs to stop the cell wall synthesis [5]. However, PBP2a has low affinity to bind with β-lactam antibiotics, which prevents β-lactam antibiotics from inhibiting cell wall synthesis and results in antibiotic resistance [6,7]. Moreover, although linezolid and vancomycin are seen as last-resort antibiotics to treat MRSA infections, the emergence of linezolid-resistant and vancomycin-resistant *S. aureus* are now presenting greater challenges to public health [8,9,10].

The high resistance of MRSA strains and the slow development of new antibiotics have prompted researchers to look for alternative therapies. One such method is a combination strategy, which aims to rescue the ability of the resistant antibiotics to inactivate bacterial strains through combining existing antibiotics [11]. For example, sulfamethoxazole and trimethoprim combination were approved by the FDA to treat MRSA skin infections [12]. There is some concern that combining antibiotics could lead, however, to an even greater degree of antibiotic resistance [13,14].

Antimicrobial peptides (AMPs) have exhibited potential as novel antimicrobials to treat infections [15]. AMPs exhibit broad-spectrum antibacterial activities and have a low potential to promote drug-resistance [15]. Negatively charged bacterial membranes attract positively charged AMPs through non-specific electrostatic forces. After attaching to bacterial membranes, AMPs form pores in the bacterial membrane, damage membrane integrity, and further cause the leakage of intracellular components, thus exhibiting antibacterial effects [16,17]. Since this type of interaction does not require specific molecular targets, it is more difficult for the bacteria to develop resistance to AMPs [18], although some researchers have found that *S. aureus* can diminish the attraction of cationic AMPs by altering the membrane charge through the peptide resistance factor MprF [19]. However, antibiotic resistance primarily develops through genetic changes in bacteria, leading to resistance against specific antibiotics, or through the horizontal transfer of resistance genes [20]. It is also quite interesting to mention that the development of bacterial resistance to one antibiotic may concurrently increase sensitivity to another antibiotic, a phenomenon known as collateral sensitivity [21].

LL37 is a cationic AMP that has 37 amino acids and plays an important antimicrobial role in the human immune system [22]. LL37 showed great potential in clinical trials, such as leading to enhanced wound healing of venous leg ulcers, especially for patients with large ulcers, and has a low potential to cause resistance [23,24,25,26]. However, the high cost of manufacturing and also the potential toxicity at high doses may limit the clinical use of LL37 [27].

The secondary structure and conformation of the AMP affects antimicrobial activity. Some regions of LL37 is less likely to form an amphipathic α-helical structure that is needed for AMPs to exhibit antimicrobial effects [28]. To have better α-helical structured peptides, the less structured fragments were cut from LL37 to obtain two more structured LL37 fragments: FK16 and FK13 [29]. FK16 and FK13 are shorter peptides derived from LL37, which will cost less to manufacture, have lower cytotoxicity, and exhibit antibacterial effects to multiple types of bacteria [29].

In this study, we determined the minimum inhibitory concentrations (MICs) of LL37, FK16, FK13, and antibiotics against three clinical MRSA strains in vitro. Further, LL37, FK16, and FK13 were combined with clinical or traditional antibiotics (vancomycin, nisin, penicillin G, ampicillin, ciprofloxacin, and linezolid) to find synergistic combinations. To study the possible mechanism for synergistic combinations, we studied the membrane permeabilizing effects of three AMPs and also studied the neutralizing process for the electrostatic interactions between AMPs with three MRSA strains. Our aims are not only to improve the effectiveness of clinical antibiotics through synergistic combinations, but also to find a possible pathway to tackle existing antibiotic-resistance. Therefore, the re-sensitization of penicillin G and ampicillin to three MRSA strains was characterized to further provide a potential antibacterial strategy based on the relationship between the uptake of antibiotics and membrane permeabilization.

## 2. Results

### 2.1. Anti-MRSA Effect of AMPs and Antibiotics

The antibacterial effects of LL37, two LL37 fragments, FK16, FK13, and 6 antibiotics were measured against three clinical MRSA strains. Vancomycin, ciprofloxacin, and linezolid exhibited antibacterial effects to MRSA strains, which was expected since these three antibiotics are also used clinically to treat MRSA infections (Table 1). LL37 did not exhibit antibacterial activity to the MRSA strains (MICs > 512 μg/mL), consistent with a previous report showing that LL37 was not effective against MRSA (MIC > 128 μM) [30]. FK13 also failed to show antibacterial effects (MICs > 512 μg/mL), but FK16 exhibited antibacterial effects to all three MRSA strains. FK16 showed better inhibitory effects to strain 33592 than strains 43300 and 43866 (16 μg/mL vs. 32 μg/mL and 64 μg/mL, respectively). This may be due to the fact that FK16 is more structured than LL37. LL37 can only form a 70–80% α-helical structure, which is known to be important for AMPs to kill bacteria [23]. Another potential factor is the influence of polyanionic components in the MHB medium, which could impact the antibacterial effects of AMPs [31]. It has been found that removing anionic inhibitors could enhance the antibacterial effects [31]. Since LL37 and FK16 were both tested in MHB medium, future research is necessary to better determine the main factors causing the differences between LL37 and FK16. Additionally, FK16 exhibits stronger antibacterial effects than FK13 because FK16 has N30, L31, and V32 residues, which are important for determining the antibacterial activity of LL37 [29]. The comparison of anti-MRSA activity between FK16 and FK13 have demonstrated that the hydrophobic C-terminus of FK16 is a crucial factor for antibacterial effects. Our results correspond to published studies that showed FK16 has lower MICs than FK13 [29,32]. 

MRSA strains have high resistance to penicillin G and ampicillin since these strains have PBP2a, which cause them to have a lower affinity to bind with penicillin-like antibiotics [6]. Nisin failed to display antibacterial effects to MRSA strains, similar to what was previously reported for other MRSA strains [33,34].

### 2.2. AMP-Antibiotic Combination Effects

FK16, FK13, and LL37 were combined with vancomycin (VAN), nisin (NS), penicillin G (PCN), ciprofloxacin (CPFX), linezolid (LZD), and ampicillin (AP) to find synergistic inhibitory combinations against three MRSA strains (Table 2). Although vancomycin and ciprofloxacin are quite effective clinically to treat MRSA infections, no synergistic combination was found when combined with three AMPs. LL37 and FK13 did not show effective anti-MRSA effects, and no synergistic combination was found when antibiotics were combined with LL37. However, there were some synergistic combinations when antibiotics were combined with FK13, which may be because of the structural differences between FK13 and LL37. Nisin only showed synergistic effects to 33592 when combined with FK16, and linezolid exhibited synergistic effects to 43300 and 33592 when combined with FK16. FK16 showed better antibacterial effects than LL37 and FK13, which may explain why FK16 had more synergistic combinations when combined with these antibiotics. 

There were more synergistic combinations among penicillin G or ampicillin when combined with AMPs. Penicillin G and ampicillin are not used clinically to treat MRSA infections since the high resistance. However, penicillin G and ampicillin showed more synergistic combinations than other antibiotics when combined with FK16 and FK13. We hypothesized that FK16 and FK13 can make MRSA strains more permeable, which allows more penicillin G and ampicillin to access MRSA and bind with PBP to achieve synergistic effects. Not surprisingly, no synergistic combinations were found when penicillin G and ampicillin were combined with LL37.

### 2.3. Membrane-Permeabilizing Effects

**Figure 1:**
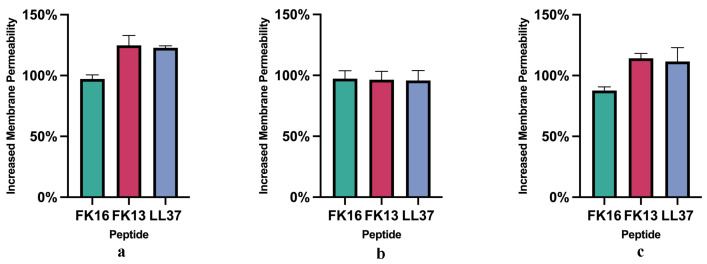
Membrane permeabilizing properties of AMPS to (**a**) MRSA ATCC (43300), (**b**) MRSA ATCC (33592), (**c**) MRSA ATCC (43866). The error bars refer to the standard deviations after three independent experiments.

To study the mechanism behind the synergistic combinations, we measured the membrane permeabilizing properties of AMPs to MRSA strains by testing the uptake of PI. PI can only enter MRSA strains with damaged membranes and can be used as an indicator of membrane integrity [35,36]. All three peptides increased membrane permeability of three MRSA strains (Figure 1). For 43300 and 43866, FK13 exhibited the strongest membrane permeabilizing properties among the three peptides at the same concentration. FK16 showed weaker membrane permeabilizing properties than FK13 and LL37. For 33592, the three peptides exhibited similar membrane permeabilizing properties to each other. Our results suggest that peptides may increase MRSA membrane permeability, potentially enhancing the access of antibiotics to their targets. 

### 2.4. Cell Wall Neutralization Process

Zeta potentials of MRSA strains were measured to show the neutralization of the negatively charged bacterial cell walls by positively charged AMPs. Teichoic acids, which are covalently linked to the peptidoglycan, cause the bacterial cell wall negatively charged [37]. Attaching to and neutralizing the negative charges caused by teichoic acids on *S. aureus* strains is necessary for cationic AMPs to exhibit antibacterial effects [38].

**Figure 2:**
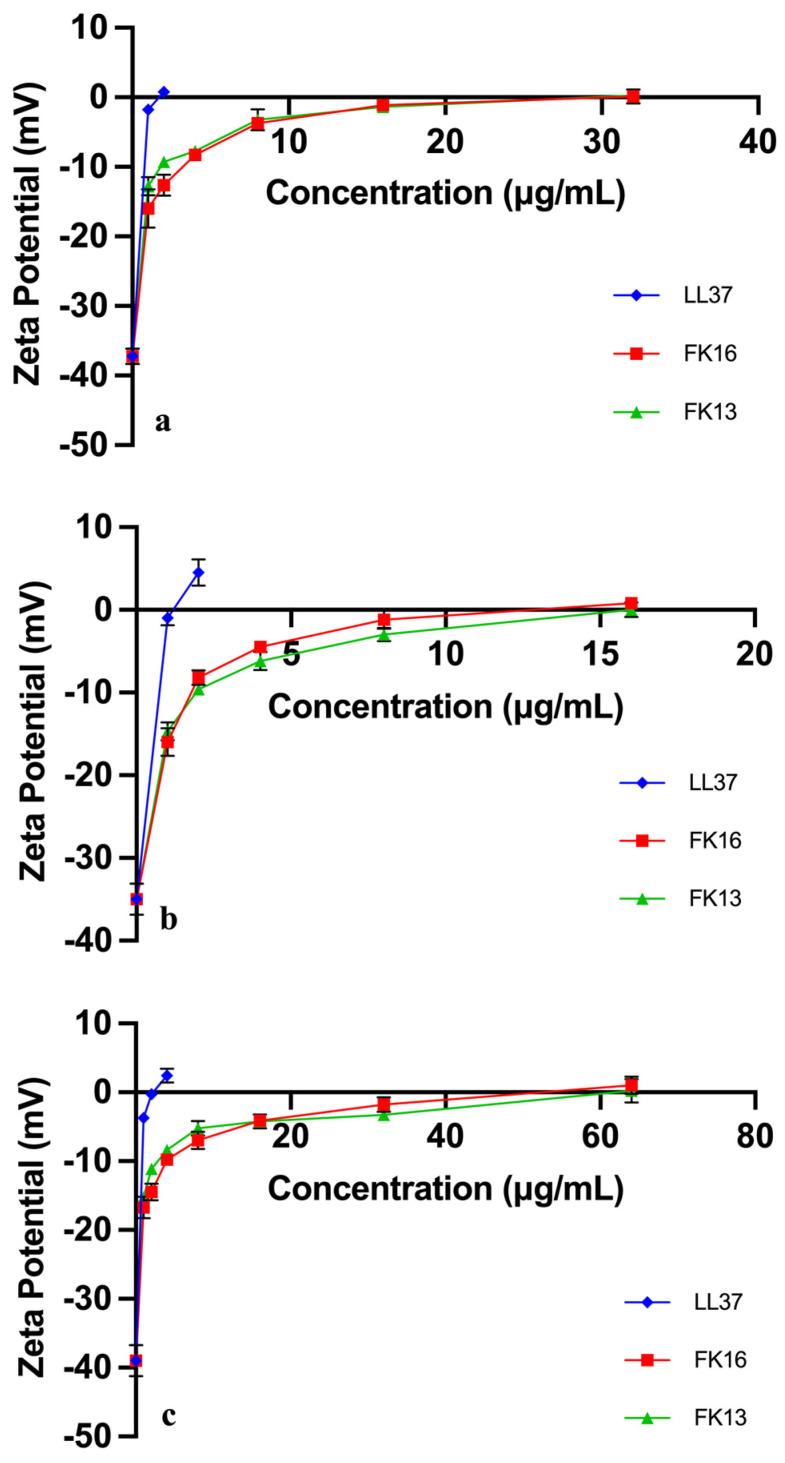
Zeta potentials of (**a**) ATCC 43300, (**b**) ATCC 33592, and (**c**) ATCC 43866. The error bars refer to the standard deviations after three independent experiments.

**Figure 3:**
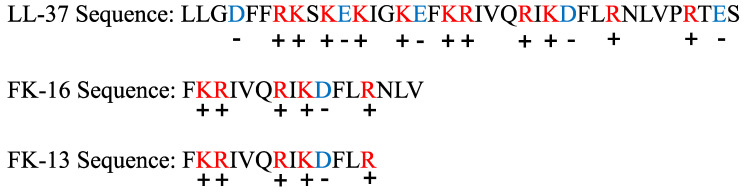
Charge distributions of LL37, FK16, and FK13. The blue letters and red letters represent the amino acids that carry negative and positive charges, respectively.

The zeta potentials for 43300, 33592, and 43866 were −37.2 mV, −35.0 mV, and −39.0 mV, respectively (Figure 2). Strain 43866 was more negatively charged compared with the other two strains, so higher concentrations of LL37, FK16, and FK13 were needed to neutralize those charges. The net charges for LL37, FK16, and FK13 are +6, +4, and +4, respectively (Figure 3). The concentrations of AMPs need to neutralize the charge scaled with the magnitude of the charge, with lower concentrations of LL37 needed to neutralize the negative charges of the three strains, and similar amounts needed for both FK16 and FK13, the latter two having the same net charges.

According to previous research, the neutralization is also related to the increase in membrane permeability [16,38]. MRSA strains need to maintain normal surface charges to ensure the integrity of their membrane, but the charge neutralization can cause alternations in membrane permeability [16,39]. 

To further decouple how charge neutralization and membrane permeability are interacting, we used ampicillin, which has no net charge, as a negative control. Ampicillin could not neutralize the negative zeta potentials of the three MRSA strains, even at a high concentration of 80 μg/mL of ampicillin (Table 3). The percent changes in the zeta potential after ampicillin exposure for 43300, 33592, and 43866 were 1.4%, 2.4%, and 2.6%, respectively. Ampicillin also failed to increase membrane permeability, even at 80 μg/mL (Table 3). The percent changes in the membrane permeability for 43300, 33592, and 43866 were 0.2%, 0.5%, and 0.3%, respectively. If the antibacterial agent lacks the ability to neutralize the negative zeta potential of the bacteria, membrane permeability is not altered [16].

### 2.5. Re-Sensitization Test of Ampicillin and Penicillin G to MRSA Strains

To validate our hypothesis regarding the promotion of ampicillin and penicillin G access to MRSA strains and the reduction of resistance through increased membrane permeability, we utilized FK16, FK13, and LL37 to treat MRSA strains and determine the new MICs for ampicillin and penicillin G.

**Figure 4:**
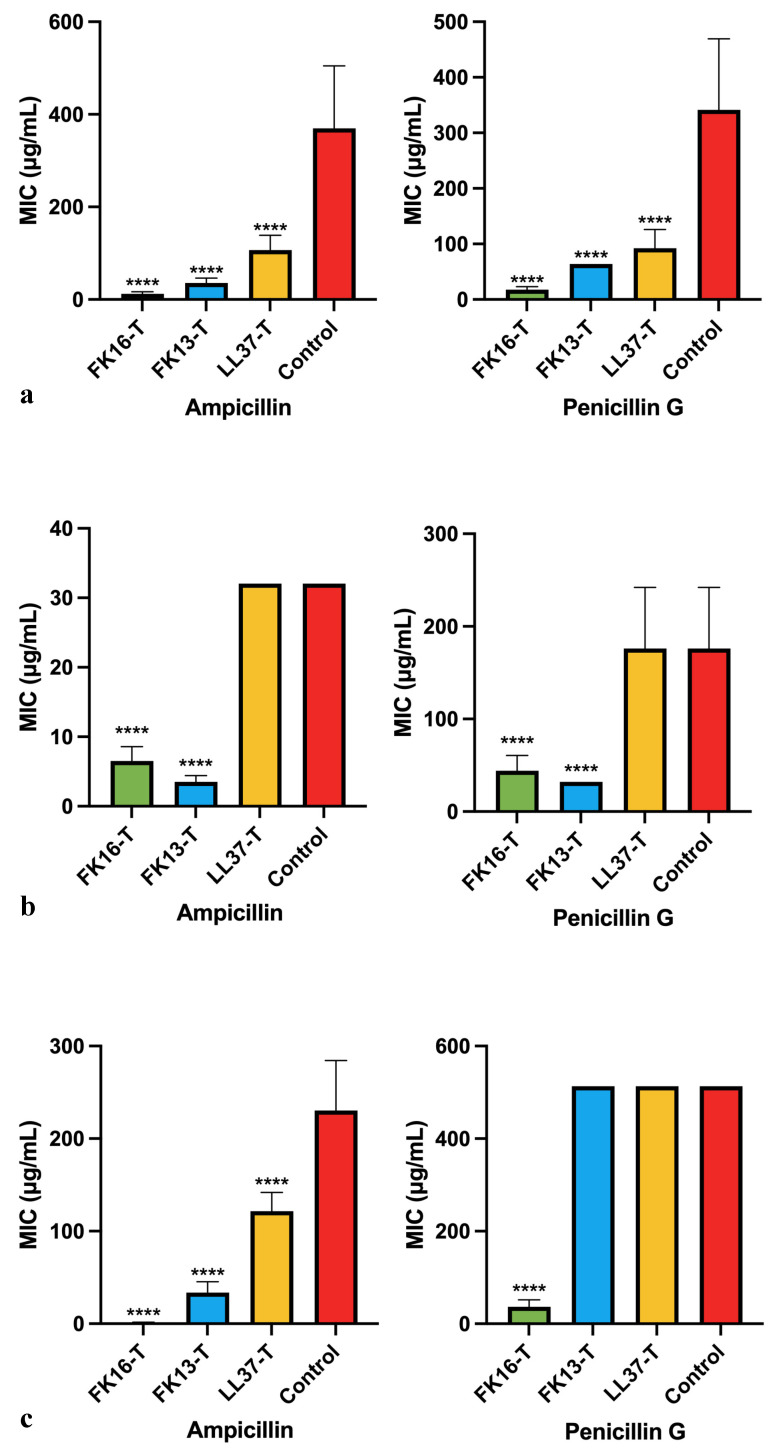
MICs of ampicillin and penicillin G against MRSA strains after peptide treatment. (**a**) MRSA 43300, (**b**) MRSA 33592, and (**c**) MRSA 43866. The control groups were given the same condition except the treatment of peptides. The error bars refer to the standard deviations after three independent experiments, and data without error bars indicate that the SD is too small to be seen. Green, blue, yellow, and red represent FK16-treated groups, FK13-treated groups, LL37-treated groups, and control groups, respectively. **** stands for *p*-value ≤ 0.0001.

All three peptides decreased the MICs of ampicillin and penicillin G when tested against 43300 (Figure 4a). The MICs of ampicillin and penicillin G decreased to 12.4 μg/mL and 17.8 μg/mL, respectively, after treatment with FK16, and the MICs of the control groups were 369.8 μg/mL and 341.3 μg/mL, respectively. FK13 and LL37 also decreased the MICs of ampicillin and penicillin G (35.6 μg/mL and 64 μg/mL vs. 106.4 μg/mL and 92.4 μg/mL), and FK13 and LL37 had especially high MICs to 43300 (MICs > 512 μg/mL). 

FK16 and FK13 decreased the MICs of ampicillin and penicillin G when tested against 33592, but LL37-treated groups showed no difference compared to the control groups (Figure 4b). FK16 decreased the MICs of ampicillin and penicillin G to 6.5 μg/mL and 44 μg/mL, and FK13 decreased the MICs of ampicillin and penicillin G to 3.5 μg/mL and 32 μg/mL, compared with the MICs of ampicillin and penicillin G in control groups (32 μg/mL vs. 176 μg/mL).

The MICs of ampicillin and penicillin G decreased to 1.2 μg/mL and 36.8 μg/mL, respectively, when tested against 43866 after treatment with FK16, compared to the control groups whose MICs were 230.4 μg/mL and >512 μg/mL (Figure 4c). FK13 and LL37 decreased the MICs of ampicillin to 33.6 μg/mL and 121.6 μg/mL, respectively. However, FK13 and LL37 did not decrease the MICs of penicillin G compared to the control groups.

FK16 exhibited the greatest potential among three AMPs since it decreased the MICs of ampicillin and penicillin G to all MRSA strains, given the high resistance of these MRSA strains to penicillin G and ampicillin (Table 4). FK13 also decreased the MICs of ampicillin and penicillin to several folds, supporting the hypothesis that increased membrane permeability enhances antibiotic accessibility to bacteria, while LL37 showed neither the highest membrane permeabilizing properties nor antibacterial effects, indicating the poorest re-sensitization effects.

## 3. Discussion

The worldwide existence of antimicrobial-resistance represents a severe threat to public health, highlighting the need for novel antimicrobials or treatments [40]. Vancomycin and linezolid are seen as last-resort antibiotics to treat MRSA infections, but the emergence of vancomycin-resistant *S. aureus* (VRSA) and linezolid-resistant *S. aureus* (LRSA) can cause a situation where even these antibiotics fail to treat drug-resistant *S. aureus* infections [41]. VRSA and LRSA strains can develop thicker cell walls to prevent the penetration of vancomycin and linezolid, and cell wall thickening has also been observed in clinical MRSA strains [42,43,44]. The efficacy of β-lactam antibiotics in the treatment of MRSA infections is diminished due to the reduced binding affinity of PBP2a in MRSA towards these antibiotics [4]. In this study, we endeavored to synergistically augment the efficacy of antibiotics against MRSA strains by incorporating AMPs to facilitate antibiotic penetration.

Antimicrobials exhibiting different target specificities on bacteria can manifest synergistic effects when used in combination against pathogens, thereby facilitating dose reduction and mitigating their individual toxicity, thus holding immense therapeutic potential [45,46]. The combination of two LL37 fragments with antibiotics exhibited several synergistic inhibitory effects to three MRSA strains. These synergistic combinations contain both clinically used antibiotics such as linezolid, and traditional antibiotics, such as penicillin G and ampicillin, which currently cannot treat MRSA infections. The peptide fragment FK16 demonstrated strong antibacterial efficacy and a higher number of synergistic interactions when combined with antibiotics, highlighting its potential as a promising antimicrobial agent for standalone or combinatorial use in combating MRSA infections.

Gram-positive bacteria contain anionic polymers called teichoic acids, which provide protection to the cell against external threats and agents. Teichoic acids influence cell permeability and integrity, contribute to bacterial cell surface charge and hydrophobicity, and affect extracellular molecule binding [47,48]. Other studies have also indicated that AMPs can form barrel-stave, carpet, toroidal-pore, or aggregate channel models upon interaction with the bacterial membrane, facilitated by electrostatic forces, thereby leading to an increase in membrane permeability [39,49,50,51,52]. Our results suggest that AMPs are prone to interacting with teichoic acids, leading to membrane perturbation. Additionally, this electrostatic interaction between AMPs and MRSA strains is predominantly influenced by the charges associated with both the antimicrobials and the bacteria.

The interaction between positively charged antimicrobials and negatively charged bacteria, driven by the electrostatic force, connects with membrane permeabilization through the neutralization process [16,38]. Bacteria need to maintain negative surface charges for normal cellular function; however, the neutralizing process can perturb membrane stability, impacting membrane integrity and increasing membrane permeability [16,38]. In this study, we observed that all three AMPs exhibited the ability to neutralize the negative zeta potentials of three MRSA strains, while increasing membrane permeability; however, it was found that ampicillin, lacking a positive charge, did not possess the capacity to neutralize negative zeta potentials or alter membrane permeability.

Increasing membrane permeability, which can be a part of a possible mechanism for synergistic combinations, may facilitate easier access of antibiotics to their targets [30]. We found that both membrane permeabilization and the antibacterial effects of AMPs are crucial factors in synergistic combinations when combined with antibiotics. FK16 both had membrane permeabilizing properties and effective antibacterial effects to three MRSA strains, which may indicate that FK16 has more synergistic combinations than other two AMPs. 

The confirmation of the relationship between increased membrane permeability and decreased resistance was demonstrated by a several-fold decrease in the MICs of ampicillin and penicillin G following treatment with three AMPs. Other studies also reported similar mechanisms. For example, the MICs of vancomycin, teicoplanin, and oxacillin to VRSA strains decreased 8- to 256-fold after treatment with WR12, an AMP with strong membrane permeabilizing properties [30]. Moreover, HAMLET (human alpha-lactalbumin made lethal to tumor cells) can enhance the susceptibility of MRSA to methicillin and vancomycin intermediate *S. aureus* (VISA) to vancomycin by facilitating increased antibiotic penetration into the bacterial cells [30,53]. These studies demonstrated the potential of antimicrobials to enhance the accessibility of antibiotics to their targets by increasing membrane permeability. Among three AMPs, only FK16 exhibited a decrease in the MICs of both ampicillin and penicillin G against three MRSA strains, thereby providing further evidence for the significance of membrane permeabilization and the inherent antibacterial properties of AMPs. 

The reason that FK16 has more antibacterial potential than LL37 and FK13 may be attributed to the structural characteristics of FK16. FK16 is more structured than LL37, which accounts for its better antibacterial efficacy, as the amphipathic α-helical secondary structure is crucial in determining the antimicrobial activity of AMPs [23,29]. Previous research has also indicated that FK16 exhibits superior antibiofilm effects compared to LL37 when tested against *S. aureus* biofilm, further illustrating the high potential of FK16 [54]. Although FK16 and FK13 are both derived from LL37, the presence of hydrophobic C-terminal residues in FK16 is crucial for determining the antibacterial activity of LL37 [29]. Our findings are consistent with previous studies indicating that FK13 exhibits weaker antibacterial effects compared to FK16 due to the absence of these three residues in FK13 [29].

In summary, the AMP-antibiotic synergistic combinations presented here provide a novel and promising strategy to combat MRSA strains. The antibacterial effects that we observed for FK16, as well as its synergistic interactions with antibiotics, highlight the significant potential of FK16 as part of a bacterial inactivation strategy. Furthermore, the favorable synergistic effects observed in FK16-ampicillin/penicillin G combinations offer the potential for the restoration of efficacy in traditional antibiotics against MRSA strains, despite the extensive drug resistance exhibited by MRSA towards penicillin-like antibiotics. The re-sensitization of MRSA strains to conventional antibiotics holds considerable significance, particularly amidst the challenges posed by the shortage of efficacious antibiotics and the protracted process of novel antibiotic development. Among the three AMPs, FK16 shows the greatest potential to become a promising antimicrobial to address the problem of antimicrobial-resistance. Further studies will need to investigate more clinically relevant conditions to move this therapy forward.

## 4. Materials and Methods

### 4.1. Ethical Statement

Ethical review and approval were not required because this study did not involve human subjects.

### 4.2. Materials

Three MRSA strains were studied in this research: ATCC 43300, ATCC 33592, and ATCC 43866, which were each stored at −80 °C. ATCC 43300 is a clinical isolate from Kansas and a representative MRSA strain for research. ATCC 33592 and ATCC 43866 are isolated from blood and urine of patients, respectively. Bacteria were cultured in Mueller Hinton broth (Sigma-Aldrich, St. Louis, MO, USA) and Mueller Hinton agar (MHA) (Hardy Diagnostics CRITERION™, Santa Maria, CA, USA). 

LL37 (LLGDFFRKSKEKIGKEFKRIVQRIKDFLRNLVPRTES), FK16 (FKRIVQRIKDFLRNLV), and FK13 (FKRIVQRIKDFLR) were purchased from Anaspec, Inc. (Fremont, CA, USA). 

Vancomycin, nisin, penicillin G, ampicillin, ciprofloxacin, and linezolid were purchased from Sigma-Aldrich (St. Louis, MO, USA). 

4-(2-hydroxyethyl) piperazine-1-ethanesulfonic acid (HEPES) buffer solution (1 M in H_2_O) and propidium iodide were also procured from Sigma-Aldrich (St. Louis, MO, USA).

### 4.3. Broth Microdilution Assay

The MICs of LL37, FK16, FK13, and antibiotics were determined based on published methods [55]. AMPs and antibiotics were dissolved in sterile deionized water and stored according to the manufacturer’s instructions. Two-fold serial dilutions of antimicrobials were added to a 96-well polypropylene microtiter plate to prepare for MIC tests. Colonies from MHA plates were incubated in MHB medium overnight in a Tissue Culture Roller Drum TC-7 (New Brunswick Scientific Co., Inc, Edison, NJ, USA) at 40 rpm and 37 °C to reach log phase. MRSA suspensions were diluted to reach the McFarland standard 0.5, yielding approximately 1.5 × 10^8^ CFU/mL. Bacterial suspensions were further diluted, and 90 μL of suspension was added to a 96-well plate to reach the final concentration of 5 × 10^5^ CFU/mL. The 96-well plate was incubated for 20 h at 37 °C, and the MICs were determined as the lowest concentrations of antimicrobials that inhibited the visible growth of MRSA [55]. The experiments were repeated three times independently.

### 4.4. Checkerboard Assay

The synergistic effects of AMP-antibiotic combinations were determined using a checkerboard assay [56,57]. 10 μL of different concentrations of AMPs and antibiotics were added to a 96-well plate to reach a 1:1 ratio of AMP and antibiotic. 80 μL of MRSA suspensions were added to a 96-well plate to reach 5 × 10^5^ CFU/mL in each well. The 96-well plate was further incubated for 20 h at 37 °C, and the synergistic combinations were determined through calculating the fractional inhibitory concentration index (FICI), which can be calculated as:

(1) $$\frac{\left(MIC\;of\;drug\;A\;in\;combination\right)}{\left(MIC\;of\;drug\;A\;alone\right)}+\frac{\left(MIC\;of\;drug\;B\;in\;combination\right)}{\left(MIC\;of\;drug\;B\;alone\right)}$$

Synergistic combinations can be determined when FICI ≤ 0.5 [58]. The experiments were repeated three times independently.

### 4.5. Propidium Iodide Uptake Assay

The membrane permeabilizing properties of AMPs to MRSA strains were determined through measuring the fluorescence of the fluorescent dye, propidium iodide (PI), using a previously published method [35,59]. Briefly, MRSA strains were incubated at 37 °C to reach the exponential growth phase, centrifuged at 4000 RPM for 5 min, and washed three times with GHEPES buffer (pH 7.25, 5 mM HEPES, 5 mM glucose). MRSA suspensions were diluted to reach 0.3 OD_600_, and 2 mL of MRSA suspensions were incubated with LL37, FK16, and FK13, with final concentrations at 50 μg/mL for 2 h at 37 °C. After incubation, MRSA suspensions were centrifuged at 8000 RPM to remove the supernatant and washed by GHEPES buffer twice. 20 μL of 1 mg/mL PI was added to MRSA suspensions and incubated in the dark for 30 min at room temperature. After incubation, MRSA suspensions, which contained PI, were added to a quartz cuvette, and the fluorescence was measured by using the F-4500 Fluorescence Spectrophotometer (Hitachi, Japan) under the following parameters: PMT voltage = 700 V, excitation wavelength = 535 nm, and emission wavelength = 620 nm. 

The increased membrane permeability was represented as the percentage of PI uptake through the equation:

PI uptake (%) = (F_AMP_ − F_C_)/(F_C_) × 100% (2)

F_AMP_ is the fluorescence of MRSA strains with PI with the addition of AMPs, and F_C_ is the fluorescence of MRSA strains with PI but without the addition of AMPs and serves as the control group. The experiments were repeated three times independently.

### 4.6. Neutralization Process

The changes in zeta potentials of MRSA strains after the addition of AMPs were determined. Briefly, MRSA strains were incubated at 37 °C to reach the exponential growth phase, centrifuged at 4000 RPM for 5 min, washed in sterilized DI water three times, and diluted to 1 × 10^6^ CFU/mL. MRSA suspensions were added to disposable folded capillary cells, and the zeta potential was determined using a Zetasizer Nano-ZS ZEN 3600 (Malvern Instruments, Inc, Westborough, MA, USA). Different concentrations of AMPs were added to bacterial suspensions, and the change in the zeta potential was recorded. Measurements were repeated three times independently.

### 4.7. Re-Sensitization Test

The re-sensitization of MRSA strains to ampicillin and penicillin G was determined after MRSA strains were treated by AMPs. MRSA strains were incubated at 37 °C to reach the exponential growth phase and diluted to 1 × 10^7^ CFU/mL. Amounts of AMPs equal to half of their MICs were incubated with MRSA suspensions at 37 °C for 1 h. The control groups were prepared at the same conditions, except that no AMPs were added. 10 μL of two-fold serial dilutions of ampicillin and penicillin G were added to a 96-well plate. After incubation, MRSA suspensions were diluted, and 90 μL of MRSA suspensions were added to a 96-well plate to reach a final concentration of 5 × 10^5^ CFU/mL. The new MICs of ampicillin and penicillin G were determined, and the folds of re-sensitization were calculated. Measurements were repeated three times independently.

## 5. Conclusions

In conclusion, the AMP-penicillin G/ampicillin synergistic combinations provide a possible approach to combat MRSA strains. The synergistic effects of FK13 and FK16 in combination with penicillin G and ampicillin may make penicillin-like antibiotics more effective in combating MRSA infections. Furthermore, the membrane-permeabilizing properties of three AMPs showed the potential to decrease the resistance of MRSA to penicillin G and ampicillin by increasing the access of antibiotics. This might be a new strategy to consider when designing antimicrobial combinations. FK16 exhibited the most effective antibacterial effects among the three AMPs, showed more synergistic combinations when combined with penicillin G and ampicillin, and also demonstrated the highest potential to decrease resistance, making it a promising antimicrobial for further study.

## Figures and Tables

**Table 1:** MICs (μg/mL) of AMPs and different antibiotics against MRSA strains.

Antimicrobials	MRSA ATCC (43300)	MRSA ATCC (33592)	MRSA ATCC (43866)
LL37	>512	>512	>512
FK16	32	16	64
FK13	>512	>512	>512
Vancomycin	1	1	1
Nisin	128	256	256
Penicillin G	128	128	>512
Ciprofloxacin	0.25	0.5	0.5
Linezolid	4	2	2
Ampicillin	128	32	256

**Table 2:** Combined activity of AMPs with antibiotics against MRSA strains.

Strain	∑ FICI						
	Combination	VAN	NS	PCN	CPFX	LZD	AP
ATCC (43300)	FK16	>0.5	>0.5	0.3125	>0.5	0.5	0.5
	FK13	>0.5	>0.5	>0.5	>0.5	<0.5	>0.5
	LL37	>0.5					
ATCC (33592)	FK16	>0.5	0.5	0.3125	>0.5	0.5	0.5
	FK13	>0.5	>0.5	<0.3125	>0.5	>0.5	<0.5
	LL37	>0.5					
ATCC (43866)	FK16	>0.5	>0.5	<0.258	>0.5	>0.5	0.252
	FK13	>0.5	>0.5	>0.5	>0.5	>0.5	0.375
	LL37	>0.5					

**Table 3:** Percent change in the zeta potential and membrane permeability with the existence of 80 μg/mL of ampicillin.

Strain	Percent Change of Zeta Potential	Percent Change of Membrane Permeability
ATCC (43300)	1.4%	0.2%
ATCC (33592)	2.4%	0.5%
ATCC (43866)	2.6%	0.3%

**Table 4:** Re-sensitization of MRSA strains to ampicillin and penicillin G after treatment with FK16, FK13, and LL37.

Strain	Fold of Re-Sensitization		
	AMPs	Ampicillin	Penicillin G
ATCC (43300)	FK16	30	20
	FK13	10	5
	LL37	3	4
ATCC (33592)	FK16	5	4
	FK13	9	6
	LL37	No sensitization effect	No sensitization effect
ATCC (43866)	FK16	192	14
	FK13	7	No sensitization effect
	LL37	2	No sensitization effect

## Data Availability

Data are available upon request.

## Abbreviations

MRSA	Methicillin-resistant *Staphylococcus aureus*
AMPs	Antimicrobial peptides
WHO	World Health Organization
*S. aureus*	*Staphylococcus aureus*
CDC	Centers for Disease Control and Prevention
PBP	Penicillin binding protein
MICs	Minimum inhibitory concentrations
VAN	Vancomycin
NS	Nisin
PCN	Penicillin G
CPFX	Ciprofloxacin
LZD	Linezolid
AP	Ampicillin
VRSA	Vancomycin-resistant *S. aureus*
LRSA	Linezolid-resistant *S. aureus*
HAMLET	Human alpha-lactalbumin made lethal to tumor cells
VISA	Vancomycin intermediate *S. aureus*
MHB	Mueller Hinton broth
MHA	Mueller Hinton agar
FICI	Fractional inhibitory concentration index
PI	Propidium iodide
